# Cause of death following revision for periprosthetic joint infection or major aseptic revision in total hip and knee arthroplasty

**DOI:** 10.5194/jbji-10-543-2025

**Published:** 2025-12-05

**Authors:** Nicolai Kjældgaard Kristensen, Per Hviid Gundtoft, Brian Elmengaard, Alma Becic Pedersen, Jeppe Lange

**Affiliations:** 1 Department of Clinical Medicine, Aarhus University, 8200 Aarhus N, Denmark; 2 University Clinic of Interdisciplinary Orthopaedic Pathways (UCOP), Elective Surgery Center, Silkeborg Regional Hospital, 8600 Silkeborg, Denmark; 3 Department of Orthopaedic Surgery, Horsens Regional Hospital, 8700 Horsens, Denmark; 4 Department of Orthopaedic Surgery, Viborg Regional Hospital, 8800 Viborg, Denmark; 5 Department of Orthopaedic Surgery, Aarhus University Hospital, 8200 Aarhus N, Denmark; 6 Department of Clinical Epidemiology, Aarhus University Hospital, 8200 Aarhus N, Denmark

## Abstract

**Background and purpose**: Periprosthetic joint infection (PJI) revisions for total knee arthroplasty (TKA) and total hip arthroplasty (THA) have increased all-cause mortality. It remains unclear whether specific causes of death contribute to this excess mortality. Our purpose was to compare the underlying causes of death in patients revised for PJI with aseptic failure and to compare THA and TKA causes of death. **Methods**: We used routinely collected data from Danish health registries. We identified 9078 patients undergoing first-time revision for PJI or aseptic failure in the Danish Hip and Knee Arthroplasty Register. PJI was primarily defined by intra-operative microbiological cultures. The causes of death were obtained from the Cause of Death Register. We used inverse probability of treatment weighting (IPTW) to adjust for confounding and calculated adjusted hazard ratios (aHRs) with a 95 % confidence interval (CI). Among 2755 deceased patients, 37 % had undergone revision for PJI and 63 % for aseptic failure. The PJI group had a higher comorbidity burden and more hip revisions but was similar in age and marital status compared to aseptic revisions. **Results**: Cancer, circulatory, and respiratory diseases were the most common causes of death in both groups. However, deaths from musculoskeletal diseases (aHR 3.04, 95 % CI: 1.67–5.56), infections (aHR 2.13, 95 % CI: 1.06–4.30), and age-related causes (aHR 2.05, 95 % CI: 1.22–3.45) were more frequent after PJI revision. **Conclusion**: The increased mortality after PJI revision appears to be multifactorial, involving a range of causes rather than a single dominant driver.

## 1 Introduction

Periprosthetic joint infection (PJI) has been established with a higher mortality compared to aseptic revisions (Kristensen et al., 2025a, b) and the age-adjusted general population (Natsuhara et al., 2019; Lum et al., 2018).

Data on the specific causes of death following PJI are nevertheless limited, and no study has directly compared these to aseptic revisions. Malignant neoplasms and circulatory disorders are described as among the most common causes of death after primary total knee arthroplasty (TKA; Hunt et al., 2017). Prange et al. (2021) identified septic shock as a leading cause of in-hospital death following PJI.

However, it remains uncertain whether the increase in mortality is primarily attributable to the infection itself or to underlying comorbidities, which could influence the PJI trajectory (Persson et al., 2023). However, it is also conceivable that patients with PJI are more susceptible to fatal infections during the PJI trajectory, while patients surviving longer after aseptic revision may face increased risk of chronic time-dependent causes of death. As a result, different patterns in cause-specific mortality may help to explain the observed mortality gap between PJI and aseptic revisions.

To our knowledge, no previous study has compared the causes of death in PJI in hip and knee revisions. Given that the mortality risk associated with PJI vs. aseptic revision differs between TKA and THA (Kristensen et al., 2025a, b), we sought to explore whether the underlying causes of death also differ between these populations.

### Purpose

We conducted this nationwide cohort study to describe and compare causes of death following revision for PJI and major aseptic revisions in THA and TKA. The secondary purpose was to examine whether specific causes of death differed between THA and TKA.

## 2 Methods

### 2.1 Study design

This nationwide register-based cohort study used routinely collected data from Danish national health databases. Denmark provides universal tax-funded healthcare to all 5.9 million residents.

We used multiple well-established Danish registries (Schmidt et al., 2014) and linked them using the unique and unchangeable personal identification number (CPR) assigned through the Civil Registration System to all nationalized citizens (Schmidt et al., 2019). The CPR number enables the up-to-date identification of the entire Danish population, including complete follow up and exact time of death.

### 2.2 Data sources

*Civil Registration System (CRS).* Established in 1968, the CRS maintains electronic records of the vital status in addition to the marital status of the Danish population. With a prevalence of missing persons around 0.3 %, the CRS is virtually complete (Schmidt et al., 2014). It provides accurate and up-to-date information on death, and with the CPR number, it allows for individual-level linkage across national registries.

*Cause of Death Register* (DAR). This monitors cause-specific mortality in Denmark, recording all deaths and classifying causes using International Classification of Diseases 10th edition (ICD-10) codes (Helweg-Larsen, 2011). Data entry is automated via the mandatory death certificate, which is linked to each deceased citizen through their CPR number. Despite limitations such as low autopsy rates and evolving coding practices, DAR remains a key resource for mortality surveillance and public health planning.

*Danish Hip Arthroplasty Register  (DHR).* The DHR has recorded all hip arthroplasties since 1995 (National Annual Report, 2023). It is a high-quality, prospectively collected dataset that is widely used for research (Gundtoft et al., 2016). Data are reported immediately by surgeons and validated annually against the Danish National Patient Registry (DNPR), with 2023 completeness of 98 % for primary and 93 % for revision procedures.

*Danish Knee Arthroplasty Register (DKR).* This register has recorded all knee arthroplasties since 1997 (Pedersen et al., 2012). It provides validated high-quality data, with 2022 completeness of 97 % for primary and 94 % for revision procedures (National Annual Report, 2023).

*Danish Microbiology dataBase (MiBa).* The MiBa has automatically collected microbiology results from all Danish departments since 2010 (Voldstedlund et al., 2014), linked via CPR numbers. These data feed into the Healthcare-Associated Infections Database (HAIBA; Gubbels, 2016). A revision was defined as a PJI if ≥2 identical positive cultures of the same bacteria out of ≥3 samples from the same revision surgery was identified. Cultures taken within 24 h before to 48 h after surgery were considered part of the same set. Contaminations were defined as only one of three samples positive or two or more of three with different pathogens, as per accepted definitions (Parvizi et al., 2018; McNally et al., 2021). This study extended microbiology inclusion beyond the standard HAIBA 1-year window until the end of the study period.

*Danish National Patient Registry (DNPR)*. This registry contains detailed information on dates, primary diagnoses, and surgical procedures, and up to 20 secondary discharge diagnoses coded according to ICD-10 (Schmidt et al., 2015).

### 2.3 Participants

Patients were included from 1 January 2010 in the PJI or aseptic cohorts from the date of their first revision surgery. Revisions for microbiologically verified PJI were included until 9 November 2023 and for surgeon-specified revision data (culture-negative PJIs) until 31 December 2020. Vital status was updated on 1 April 2025, extending follow up from our previous studies (Kristensen et al., 2025a, b).

This study includes patients > 40 years of age at the time of primary TKA or THA, who had their first revision surgery due to either PJI or major aseptic causes following primary TKA or THA for osteoarthritis. For patients with bilateral revisions, only the first was included; contralateral revisions were excluded, as cause of death is a person-level outcome.

### 2.4 PJI revision cohort

The PJI cohort included patients identified via DKR, DHR, and HAIBA. PJI was considered confirmed if the procedure in DKR and DHR was recorded as a deep infection by the surgeon or if positive cultures were recorded in HAIBA for any given TKA and THA revision. Revisions with clinical suspicion but no microbiological confirmation were classified as “culture-negative PJI”. The PJI cohort included all revision types including one stage, two stage, and DAIR (debridement, antibiotics, and implant retention). This method has been validated as a reliable way to identify PJI revisions in Danish registries (Anneberg et al., 2024; Gundtoft, 2017).

### 2.5 Aseptic revisions cohort

The aseptic cohort included patients undergoing first-time major aseptic revision recorded in DKR or DHR. An aseptic revision was defined as a procedure where the operating surgeon recorded no suspicion of infection and where intra-operative samples registered in HAIBA did not meet the criteria for PJI. Major aseptic revision was defined as the exchange of metal components interfacing with bone, excluding minor procedures such as the isolated exchange of polyethylene spacers.

### 2.6 Variables

To address potential confounders between revision type and mortality, a directed acyclic graph (DAG) model was applied. Age at revision, gender, and weight were retrieved from DKR and DHR. We used weight instead of body mass index (BMI), as height data were largely missing and the missingness was unevenly distributed between hip and knee revisions. Comorbidity was assessed using the Charlson comorbidity index (CCI), extracted from DNPR and categorized as low (0), medium (1–2), or high ( ≥3 ) based on status at primary arthroplasty. Marital status at revision (single, cohabiting, or never married) and vital status were obtained from CRS.

To comply with national reporting guidelines, which prohibit the disclosure of data at the individual level, only ICD-10 chapters with ≥5 deaths were included in the final analysis, which was done separately for the PJI and aseptic cohorts.

### 2.7 Outcome

The primary outcome was the cause of death in patients undergoing revision for PJI or major aseptic failure. Cause of death was identified using DAR and linked to revision data (DKR, DHR) and HAIBA. DAR records the causes of death using ICD-10 codes, which are then grouped into ICD-10 chapters (WHO, 2019). The underlying cause of death, as opposed to the immediate cause, was used in the analysis, as this is more representative of the patients' trajectory than the last condition that caused the patient to decease.

### 2.8 Data analyses

Baseline characteristics were summarized using means, counts, and percentages, with standard deviations (SDs) where relevant. Continuous variables were assessed for skewness via visual inspection and Q – Q plots. CCI was categorized as low, medium, or high based on predefined thresholds.

Causes of death were analyzed for the total cohort and separately for TKA and THA.

The adjusted analysis used IPTW to account for measured confounders (Chesnaye et al., 2022). IPTW was preferred over multivariable regression due to its theoretical ability to reduce treatment selection bias by creating a balanced pseudo-population. Propensity scores were estimated using logistic regression as the predicted probability of death, given confounders as described in variables. IPT weights were stabilized using marginal probability of treatment assignment to improve precision. Covariate balance between cohorts was assessed using standardized mean differences, with <0.1 indicating sufficient balance. Using IPTW-adjusted data, adjusted hazard ratios were calculated via Cox proportional hazards regression. The proportional hazards assumptions were tested using Schoenfeld residuals and visual inspection, confirming no violations. The main investigator had full access to pseudonymized data. Data cleaning included cross-validation across registries.

Analyses and data management were performed using R version 4.0.2 (R Foundation for Statistical Computing). The study is reported in accordance with the RECORD (REporting of studies Conducted using Observational Routinely-collected Data) guidelines. All estimates are reported with 95 % confidence intervals unless otherwise stated, and means are presented with standard deviations in parentheses.

### 2.9 Patient characteristics

Among 9078 first-time TKA and THA revisions (both PJI and aseptic), 2755 patients died during follow up, with 37 % ( n=1007 ) in the PJI group and 63 % ( n=1748 ) in the aseptic revision group. There were 266 patients in the PJI group who were culture negative (Kristensen et al., 2025a, b). Patients in the PJI group had a higher comorbidity burden, with a mean CCI of 1.36 (SD: 1.91) compared to 0.93 (SD: 1.42) in the aseptic group ( p<0.001 ). Men were more common in the PJI group compared to women (52 % vs. 44 %, p<0.001 ). Body weight was slightly higher in the PJI group at 88 (SD: 22) kg, compared to 84 (SD: 19) kg in the aseptic group ( p<0.001 ). Demographic and baseline differences are shown in Table 1.

**Table 1:** Demographic and clinical characteristics of deceased patients following first-time revision for PJI or aseptic failure.

Deceased patient demography	Aseptic	PJI	P value^c^
$N_{1}$	1748	1007	
Joint_1_			<0.001
THA	958 (55 %)	623 (62 %)	
TKA	790 (45 %)	384 (38 %)	
Age at primary^b^	74 (9)	74 (9)	0.8
Age at revision^b^	75 (9)	75 (9)	0.2
Gender_1_		<0.001	
Male	764 (44 %)	520 (52 %)	
Charlson comorbidity index^b^	0.93 (1.42)	1.36 (1.91)	<0.001
Charlson comorbidity index category^a^			<0.001
1 (CCI = 0)	951 (54 %)	451 (45 %)	
2 (CCI = 1–2)	597 (34 %)	372 (37 %)	
3 (CCI > 3)	200 (11 %)	184 (18 %)	
Follow up in years^b^	6.0 (3.5)	5.1 (3.5)	<0.001
Weight, kg_2_	84 (19)	88 (22)	<0.001
Unknown weight^a^	775	573	
Marital status_1_			0.4
Cohab	1050 (60 %)	602 (60 %)	
Single	537 (31 %)	296 (29 %)	
Never married	161 (9.2 %)	109 (11 %)	

^a^ n (%); ^b^ mean (SD); ^c^ Pearson's $\chi^{2}$ test; Kruskal–Wallis rank sum test. P values reflect differences between deceased patients in the PJI and aseptic revision groups. The “ N ” row indicates the number of deceased patients in each group included in the analysis.

## 3 Results

**Figure 1:**
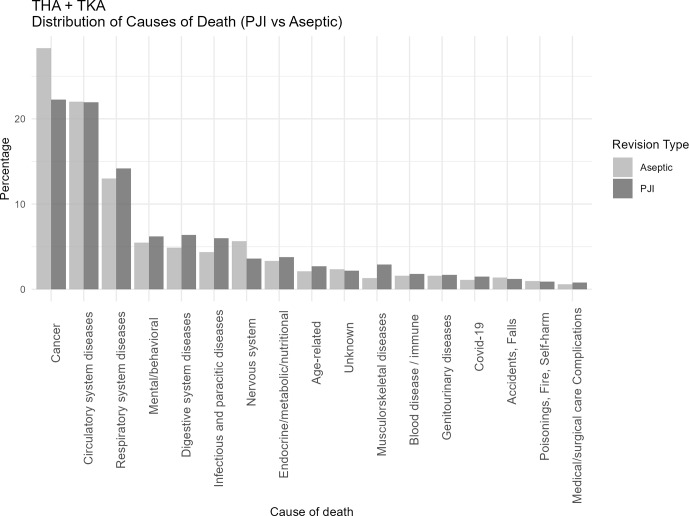
Distribution of causes of death categorized according to ICD-10 chapters among patients undergoing revision total hip or knee arthroplasty for periprosthetic joint infection (PJI) or major aseptic failure. Only chapters containing five or more cases are presented. For each revision type, the relative distribution is shown, with all causes summing to 100 % in the PJI and aseptic revision groups, respectively. Each death only occurs in one cause of death. Abbreviations: PJI = periprosthetic joint infection; Aseptic = major aseptic revision; TKA = total knee arthroplasty; THA = total hip arthroplasty.

The overall distribution of causes of death for the entire cohort (THA + TKA) was not notably different from the PJI and aseptic revision cohorts (Fig. 1). In both groups (PJI and aseptic), the most common causes of death ICD-10 chapters were cancer (26 %), circulatory system diseases (22 %), and respiratory diseases (13 %). However, the PJI group showed a higher proportion of deaths from infectious (5.9 % [95 % CI: 4.5–7.6]), digestive (6.4 % [5.0–8.2]), and musculoskeletal diseases (2.9 % [2.0–4.1]) compared to the aseptic group (4.3 % [3.4–5.4], 4.8 % [3.9–6.0], and 1.3 % [0.8–2.0], respectively). Conversely, cancer (22.3 % [19.8–25.0] vs. 28.2 % [26.1–30.3]) and nervous-system-related deaths (3.6 % [2.5–4.9] vs. 5.6 % [4.6–6.8]) were more frequent in the aseptic group.

**Figure 2:**
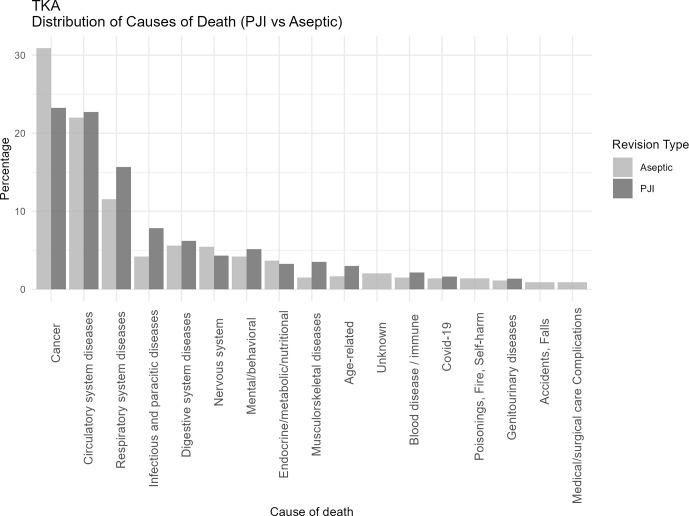
Distribution of causes of death categorized according to ICD-10 chapters among patients undergoing revision total knee arthroplasty for PJI or major aseptic failure. Only chapters containing five or more cases are presented. For each revision type, the relative distribution is shown, with all causes summing to 100 % in the PJI and aseptic revision groups, respectively. Abbreviations: PJI = periprosthetic joint infection; Aseptic = major aseptic revision; TKA = total knee arthroplasty.

**Figure 3:**
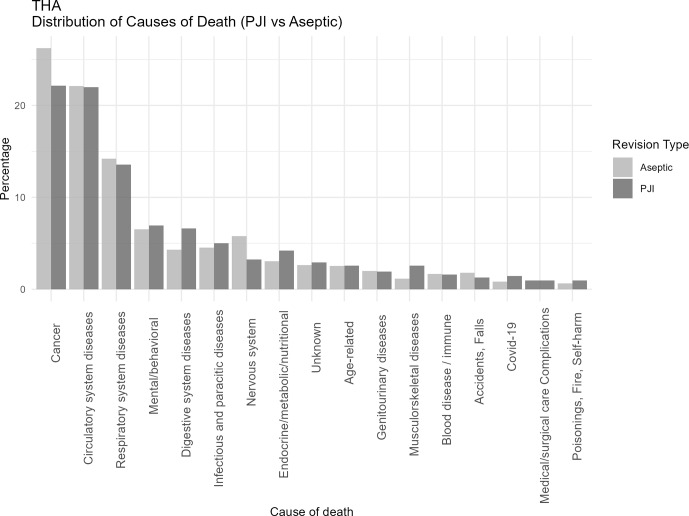
Distribution of causes of death categorized according to ICD-10 chapters among patients undergoing revision total hip arthroplasty for PJI or major aseptic failure. Only chapters containing five or more cases are presented. For each revision type, the relative distribution is shown, with all causes summing to 100 % in the PJI and aseptic revision groups, respectively. Abbreviations: PJI = periprosthetic joint infection; Aseptic = major aseptic revision; THA = total hip arthroplasty.

Table 2 presents the most frequently reported specific causes of death (as recorded on death certificates) corresponding to the broader ICD-10 chapters displayed in Figs. 1–3.

**Table 2:** Examples of what the chapters of cause of death contain in this cohort.

Infectious	Sepsis, hepatitis
Cancer	Malignant neoplasm and of lung, prostate, breast, colon
Blood	Myelodysplastic syndrome, anemia
Endocrine	Diabetes mellitus type 2 with multiple complications, dehydration
Mental/behavioral	Dementia, alcohol dependence, vascular dementia
Nervous system	Parkinson's, Alzheimer's
Circulatory diseases	Stroke, heart failure, chronic ischemic heart failure, acute myocardial infarction, atrial
	rhythm diseases, aortic valve stenosis
Respiratory	Chronic obstructive pulmonary disease, bacterial pneumonia, pneumonia
Digestive system	Alcoholic cirrhosis of liver, gastrointestinal hemorrhage
Musculoskeletal	Rheumatoid arthritis, osteoarthrosis
Genitourinary	Urinary tract infection, chronic kidney disease
Age-related/unknown	Unknown, senility, unattended death
COVID-19	COVID-19 confirmed by lab
Accidents, falls	Slipping, tripping, falls, same level, different level
Other exposures	Accidental exposure unspecified, accidental poisoning by drugs, self-harm
Medical and surgical	Surgical operation with implant of artificial internal device as the cause of abnormal
complications	reaction of the patient

After adjustments using the IPTW method, as shown in Table 3, the PJI group demonstrated significantly higher mortality in several cause of death ICD-10 chapters. These included musculoskeletal ( P<0.01 ) and infectious diseases ( P<0.01 ), COVID-19 ( P=0.03 ), age-related causes ( P<0.01 ), digestive system diseases ( P=0.01 ), mental and behavioral disorders ( P<0.01 ), respiratory diseases ( P<0.01 ), and circulatory diseases ( P<0.01 ). No significant differences were observed in the remaining ICD-10 chapters. The differences between revision types in the adjusted analysis were observed in causes related to infection, aging, and chronic disease.

**Table 3:** IPTW-adjusted hazard ratios for cause-specific death in PJI vs. aseptic revisions. Hazard ratios (HRs) with 95 % confidence intervals (CIs) for cause-specific mortality were estimated using Cox models with stabilized IPTW, comparing PJI and aseptic revisions. Follow-up time was measured from revision to death or censoring. The number of deaths ( N ) in each category is reported for both groups combined. Statistically significant results are highlighted in bold.

Cause of death categories	HR	95 % CI	P value	N deaths
**Musculoskeletal diseases**	**3.04**	**1.67–5.56**	< **0.01**	**52**
**COVID-19**	**2.13**	**1.06–4.3**	**0.03**	**34**
**Infectious and parasitic diseases**	**2.13**	**1.48–3.08**	< **0.01**	**136**
**Age-related**	**2.05**	**1.22–3.45**	< **0.01**	**64**
Medical/surgical care complications	2	0.78–5.15	0.15	18
**Digestive system diseases**	**1.77**	**1.25–2.5**	**0.01**	**149**
**Blood disease/immune**	**1.7**	**0.91–3.16**	**0.09**	**46**
**Mental/behavioral**	**1.65**	**1.17–2.33**	< **0.01**	**157**
**Respiratory system diseases**	**1.45**	**1.16–1.83**	< **0.01**	**368**
Genitourinary diseases	1.36	0.68–2.7	0.38	45
**Circulatory system diseases**	**1.34**	**1.12–1.61**	< **0.01**	**603**
Endocrine/metabolic/nutritional	1.27	0.82–1.98	0.27	96
Cancer	1.15	0.96–1.36	0.12	715
Poisonings, fire, self-harm	1.14	0.49–2.65	0.77	26
Accidents, falls	1.07	0.52–2.22	0.85	36
Nervous system	1.04	0.69–1.57	0.84	134
Unknown	0.97	0.57–1.65	0.90	63

When comparing joint-specific THA and TKA revision cohorts (Figs. 2 and 3), the overall distribution of cause-of-death categories appeared comparable, with similar patterns observed across all causes of death. Respiratory deaths were notably more frequent in the TKA-PJI group vs. TKA aseptic with 15 % (12 to 19.6) vs. 11.6 % (9.5 to 14.0), while digestive with TKA-PJI of 6.7 % (4.9 to 9.0) vs. TKA-aseptic of 4.2 % (3.11 to 5.8), and endocrine-related deaths with TKA-PJI of 4.1 % (2.8 to 6.1) vs. TKA-aseptic of 3 % (2.1 to 4.4) were more common in THA-PJI patients. Infectious causes were elevated in both, particularly in TKA-PJI compared to the aseptic cohort.

## 4 Discussion

In this study, we wanted to examine whether the registered cause of death could help to identify patterns that might explain the higher mortality following revision for PJI compared to aseptic failure in hip and knee arthroplasty.

We found the distribution of registered ICD-10 causes of death to be similar between revision types, with cancer, and circulatory and respiratory diseases being the most frequent in both groups. We could not identify any distinct patterns in the available dataset.

However, there were several causes of death, including infectious, musculoskeletal, digestive, respiratory, and circulatory diseases that were more commonly reported in PJI revisions compared to aseptic revisions. Our findings suggest that the excess mortality found after PJI revisions are not due to any single cause but rather indicates an elevated risk across multiple common fatal conditions. This could suggest that the PJI and its treatment impose a physiological burden, reducing patients' resilience to conditions that might otherwise be survivable. The clinical implication of this is important as it gives attention to broader systemic vulnerability beyond the infection itself. This may warrant involving internal medicine and infectious disease specialists to support patient management in a multi-disciplinary team setting. Despite PJI patients having a higher baseline comorbidity as measured by the Charlson comorbidity index, the increased mortality risk persists even when adjusting for these measured confounders in advanced statistical methods (Kristensen et al., 2025a, b).

Drain et al. (2022) similarly found no single cause explaining higher mortality in septic revision TKA. Rather, risk appeared to be tied to pre-operative frailty and the disease process itself. The septic cohort had higher pre-operative morbidity and experienced greater increases in CCI post-operatively. Although mortality rates were significantly higher in the septic group at multiple time points, no specific cause of death differed between the cohorts, mirroring the results of our study. Bozic et al. (2012) identified comorbidities such as heart failure, anemia, and diabetes as predictors of mortality after primary TKA, underscoring the impact of patient factors even if the context differs from the revision settings. However, differences in patient populations, surgical context, and outcome measures make direct comparisons impossible.

Persson et al. (2023) concluded that excess mortality following THA-PJI was largely attributable to comorbidities. To reduce selection bias, they compared PJI revisions to a broader group of non-infected revisions. However, the Swedish registry used has a sensitivity of 67 % for identifying PJI revisions when microbiological confirmation was not applied (Lindgren et al., 2014), and their comparator group was heterogeneous, potentially underestimating the mortality risk. In our study, we addressed these limitations by using microbiologically confirmed infections and by restricting the comparator group to surgically similar aseptic revisions.

Despite methodological differences, our findings align with previous estimates and support the notion that excess mortality after PJI revision reflects broader systemic vulnerability, not fully accounted for by comorbidity as measured by the Charlson comorbidity index.

### 4.1 Potential limitations

The quality of the data on causes of death relies mainly upon the correctness of the physicians' notification (Helweg-Larsen, 2011). The risk of misclassification in DAR is not negligible, as hospital deaths are often certified by junior doctors who may lack full insight into the clinical course. Furthermore, death certification may occur outside of orthopedic settings, where the revision procedure is unlikely to be a primary consideration. However, this misclassification is likely non-differential between the PJI and aseptic groups, which would bias the results toward the null and potentially underestimate true differences in cause-specific mortality.

We chose to analyze the underlying cause of death as it is the most widely accepted approach in epidemiological mortality research. The underlying cause is defined as the disease or condition that initiated the sequence of events leading to death. In contrast, immediate or main causes of death often reflect terminal events (e.g., cardiac arrest or sepsis), which may not be causally linked to the prior infection or revision procedure, particularly when occurring months or years later. By focusing on the underlying cause, we aimed to capture deaths in which the certifying physician considered the PJI or its consequences to have played a contributory role.

We used weight instead of BMI due to substantial missing height data, which was unevenly distributed between hip and knee revisions. While BMI is more commonly used, weight remains a relevant predictor in arthroplasty (Mulhall et al., 2007). The average weight was, however, without any clinically relevant difference.

Although adjustments with the propensity model IPTW have been made for variables available in the cohort, residual confounding cannot be excluded, hence a potential impact could exist. While we adjusted for comorbidities using CCI derived from the DNPR, it is important to note that the CCI does not capture all the relevant aspects of a patient's health. Conditions such as frailty, functional impairment, and less severe but clinically meaningful diseases may not be reflected in CCI scores. This limitation may have led to residual confounding. Furthermore, restricting the analysis to the first revision each patient underwent ensured one observation per individual but may have excluded subsequent revisions, which are potentially associated with higher mortality. As a result, the overall mortality risk may have been underestimated.

Selection bias is a potential limitation when comparing outcomes between PJI and aseptic revision patients. Patients selected for revision due to PJI may differ systematically from those undergoing aseptic revisions in various ways, not fully captured by recorded comorbidities or demographics. These selection mechanisms could bias estimates of excess mortality in either direction, depending on the clinical setting and healthcare practice.

Due to data availability, culture-negative PJIs from January 2021 to November 2023 were not captured, potentially leading to underrepresentation in the later study period.

### 4.2 External validity

The study was conducted in a Danish healthcare setting, which may differ from non-Scandinavian countries in terms of population health, access to care, and post-operative rehabilitation practices. As the Danish universal healthcare system provides free and equal access to diagnostics, treatment, and surgery, this may strengthen the internal validity of the findings. However, the external validity may be limited in settings with different healthcare systems or resource constraints.

### 4.3 Ethical considerations

This study was conducted in accordance with the ethical principles outlined in the Declaration of Helsinki. Approval was obtained from the relevant institutional review boards. Patient confidentiality and data security were rigorously maintained throughout the study, and all analyses adhered to applicable data protection regulations. The study is registered with the appropriate regional authority with case ID 1-16-02-160-22. This research did not receive any specific grant from funding agencies in the public, commercial, or not-for-profit sectors.

## 5 Conclusion

This nationwide cohort study found that the excess mortality following PJI revision appears multifactorial and not attributable to a single reported cause of death.

While infectious causes were more frequent in the PJI revision group, no single cause of death accounted for the excess mortality between PJI and aseptic revisions. These findings suggest that the elevated mortality following PJI revision is likely multifactorial, reflecting a broader systemic vulnerability rather than a single pathophysiological mechanism or merely the presence of pre-existing comorbidities. The results highlight the need for targeted peri-operative strategies and long-term follow up in this high-risk patient population.

### Declaration of generative AI and AI-assisted technologies in the writing process

During the preparation of this work, the authors used ChatGPT to enhance the language and improve clarity. After using this service, the authors reviewed and edited the content as needed. They take full responsibility for the content of the publication.
